# The Size Effect of TiO_2_ Hollow Microspheres on Photovoltaic Performance of ZnS/CdS Quantum Dots Sensitized Solar Cell

**DOI:** 10.3390/ma12101583

**Published:** 2019-05-15

**Authors:** Zhen Li, Libo Yu

**Affiliations:** 1 Institute of Flexible Composite Materials, College of Chemistry and Chemical Engineering, Hexi University, Zhangye 734000, China; lizhen@hxu.edu.cn; 2 Key Laboratory of Hexi Corridor Resources Utilization of Gansu, Hexi University, Zhangye 734000, China

**Keywords:** titanium dioxide, hollow microspheres, quantum dots sensitized solar cells

## Abstract

Size controllable TiO_2_ hollow microspheres (HMS) were synthesized by a carbonaceous spheres (CS) template method. Based on TiO_2_ HMS, the ZnS/CdS quantum dots (QDs) were loaded to form a ZnS/CdS@TiO_2_ HMS photoanode for quantum dots sensitized solar cell (QDSSC). The size effects of TiO_2_ HMS on photovoltaic performance were investigated, and showed that TiO_2_ HMS with sizes ~560 nm produced the best short-circuit current density (J_sc_) of 8.02 mA cm^−2^ and highest power conversion efficiency (PCE) of 1.83%, showing a better photovoltaic performance than any other QDSSCs based on TiO_2_ HMS with size ~330 nm, ~400 nm, and ~700 nm. The improvement of photovoltaic performance based on ~560 nm TiO_2_ HMS which can be ascribed to the enhanced light harvesting efficiency caused by multiple light reflection and strong light scattering of TiO_2_ HMS. The ultraviolet-visible (UV-vis) spectra and incident photo to the current conversion efficiency (IPCE) test results confirmed that the size of TiO_2_ HMS has an obvious effect on light harvesting efficiency. A further application of ~560 nm TiO_2_ HMS in ZnS/PbS/CdS QDSSC can improve the PCE to 2.73%, showing that TiO_2_ HMS has wide applicability in the design of QDSSCs.

## 1. Introduction

Hollow micro-spherical structures of metal oxide materials have attracted much attention due to their broad range of applications in vehicles for the controlled release of substances, catalysis, photonic devices, and energy [1,2]. Especially in energy-related systems, various metal oxide hollow microspheres (HMS) such as TiO_2_, ZnO, and Fe_2_O_3_ have been used in photocatalysis, dye or quantum dots sensitized solar cells, lithium ion batteries, sensors, and supercapacitors [3,4,5]. It is well known that the hollow spherical structure is capable of providing an enhanced surface-to-volume ratio and a reduced transport length for both mass and charge transport [6,7,8]. Among these types of metal oxide HMS, TiO_2_ HMS has become one of the hot topics in research, particularly in solar energy conversion devices such as dye sensitized solar cells (DSSCs) or quantum dots sensitized solar cells (QDSSCs).

TiO_2_ can function as a supporting architecture in DSSCs or QDSSCs because of some intrinsic material characteristics, including chemical and optical stability, low fabrication cost, high electron mobility, and matched band alignment to sensitizers [9,10,11,12]. In addition, hollow spherical structures of TiO_2_ are believed to have the advantages of low bulk density, high specific surface area, and good surface permeability in DSSCs or QDSSCs [13]. Although various shapes of TiO_2_ structures such as nanotubes, nanorods, mesoporous beads, and microspheres have been used to tailor the optical and electronic properties of photoanodes in DSSCs or QDSSCs [8,14,15,16]. TiO_2_ HMS is believed to have outstanding advantages in enhancing light harvesting efficiency due to the multiple reflections of incident light within the interior cavity of HMS.

Until now, several approaches such as the Ostwald-ripening formation mechanism and template-assisted method have been developed for the synthesis of TiO_2_ HMS [17]. The Ostwald-ripening formation mechanism is widely reported in the solvothermal or hydrothermal process to produce TiO_2_ HMS: however, getting the size uniform to TiO_2_ HMS remains a challenge by this mechanism. The template-assisted method seems better for the controllable synthesis of HMS [18,19]. Carbonaceous spheres are preferred due to their functional groups on the surface and their easy removal by the heating process in air [20]. Furthermore, the size of TiO_2_ HMS from small to big can be well tuned using carbonaceous sphere templates of different sizes. In DSSCs or QDSSCs, the TiO_2_ HMS as the architecture of photoanodes shows potential in improving photovoltaic performance. Yong et al. reported a type of DSSC with a power conversion efficiency of 9.50% based on TiO_2_ HMS with a size around 500–700 nm [21]; their TiO_2_ HMS can generate a strong light scattering, leading to better light harvesting efficiency than TiO_2_ nanoparticles. A similar conclusion also was echoed in DSSCs by Jiang et al. using their Au@TiO_2_ HMS with sizes around ~500 nm, their DSSC achieved a power conversion efficiency of 7.81% which obtained a 30% increment of power conversion efficiency in comparison to conventional DSSCs fabricated with P25 TiO_2_ photoanodes [22]. However, these investigations of TiO_2_ HMS in solar cells almost focused on comparing the conventional TiO_2_ nanoparticles. The systematic research about the size effect of TiO_2_ HMS on photovoltaic performance seems to have been neglected. In fact, the size effect is important because it is generally known that strong light scattering should occur only when the size of a sphere is comparable to the wavelength of incidental light [23,24,25]. Therefore, the different sizes of TiO_2_ HMS have been synthesized by us to serve as supporting architectures in ZnS/CdS QDSSCs, and their size effect on photovoltaic performance has been investigated. To our best knowledge, this kind of inspiration reveals that the size effect of TiO_2_ HMS on QDSSCs’ performance has not been widely reported and may provide insights into the design of highly efficient QDSSCs from the perspective of supporting architectures.

## 2. Materials and Methods

### 2.1. Materials

Sucrose, titanium tetrachloride, ethylcellulose, terpineol, ethanol, cadmium nitrate, zinc nitrate, sodium sulfide, and sulfur powder were purchased from Aladdin Co., Ltd.; all these solvents and chemicals were of analytical grade and used without further purification. The fluorine-doped tin oxide (FTO) conductive glass was purchased from Opvtech Co., Ltd. Deionized water was used throughout the experiments.

### 2.2. Preparations of Carbonaceous Spheres (CS) Template

The carbonaceous spheres (CS) template were hydrothermally synthesized with sucrose aqueous solution similar to the previous report in [13]. In a typical synthetic route, sucrose aqueous solution was sealed in a Teflon stainless autoclave for 8 h at 180 °C. Then, the acquired black precipitate was washed with deionized water three times and dried in an oven for 12 h at 80 °C. In order to get CS templates with different sizes, the concentrations of sucrose solution were adjusted to 0.5 M, 0.75 M, 1.0 M, and 1.5 M, respectively.

### 2.3. Size Controllable Synthesis of TiO_2_ Hollow Microspheres (HMS)

Based on CS templates, TiO_2_ hollow microspheres (HMS) were further synthesized. Briefly, 5 g CS template were dispersed in 3 M TiCl_4_ aqueous solution (100 mL) which was pre-prepared by dropping TiCl_4_ into a deionized ice bath. After 10 min, magnetic stirring and a subsequent 20 min ultrasonication, the mixture was aged for 6 h at room temperature. The mixture was filtered and washed three times, and was transferred into oven drying at 80 °C for 12 h. The dried black product was heated in Muffle furnace with rate of 4 °C/min to 500 °C, and holding at this temperature for 4 h, producing white TiO_2_ HMS powder. The TiO_2_ HMS with a size from small to big were controllably synthesized using different sizes of CS template.

### 2.4. Fabrication of ZnS/CdS@TiO_2_ HMS Photoanode

The first step is to construct TiO_2_ HMS on FTO glass as follows. The viscous mixture containing TiO_2_ HMS powder (3.0 g), ethylcellulose (0.5 g), ethanol (5 mL), and terpineol (10 mL) was under magnetic stirring for 1 h, and was used as a paste to cover the conductive surface of FTO glass (2.0 cm × 1.5 cm) by the doctor-blade technique; the thickness of the TiO_2_ HMS film on FTO glass was controlled to be ~15 μm, and the active area was adjusted to 0.25 cm^2^ by a spacer with corresponding area and thickness; after being dried at room temperature, the photoanode was heated to 450 °C for 1 h to remove any organic residuals, forming TiO_2_ HMS photoanodes.

The second step is the fabrication of ZnS/CdS@TiO_2_ HMS photoanode by the classic successive ionic layer adsorption and reaction (SILAR) method [26,27]. In general, 0.05 M cadmium nitrate solution was prepared by mixing methanol and deionized water (7:1, V/V) as a Cd^2+^ source, and 0.05 M sodium sulfide solution was prepared with a mixed solution of methanol and deionized water (1:1, V/V) as a S^2−^ source. The TiO_2_ HMS photoanode was first immersed in Cd^2+^ solution for 30 s, washed with methanol and dried by air gun. Then dipping the photoanode into S^2−^ solution for another 30 s, washed and dried again. These two procedures were defined as one SILAR cycle, and 8 SILAR cycles were repeated on TiO_2_ HMS photoanode to guarantee enough CdS QDs loadings. Similarly, two SILAR cycles of ZnS were covered on CdS QDs as passivation layers to form ZnS/CdS@TiO_2_ HMS photoanodes with the purpose to enhance the stability of the photoanode [28]. In order to investigate the size effect of TiO_2_ HMS on photovoltaic performance, identical SILAR cycles of ZnS/CdS QDs were loaded on TiO_2_ photoanodes which were constructed by different sizes of TiO_2_ HMS.

### 2.5. Solar Cell Assembly

Cu_2_S film formed on a brass sheet was employed as a counter electrode, which was prepared according to a previous report [29]. Brass foil with size of 2.0 cm × 1.5 cm and thickness of 0.3 mm was first etched in HCl solution at 80 °C for 15 min to the exposure of copper. Following a wash with deionized water and drying by air gun, the etched brass foil was immersed in polysulfide solution which is composed of 1M sodium sulfide and 1M sulfur in deionized water, causing the exposure surface of etched brass foil to turn black. This color change demonstrated the formation of Cu_2_S. To fabricate QDSSC, the ZnS/CdS@TiO_2_ HMS photoanode and Cu_2_S counter electrode were clipped together like a sandwich for the open I-V test, to which one drop of polysulfide electrolyte was filled between the photoanode and the counter electrode as a scavenger of holes. The QDSSCs based on TiO_2_ HMS with different sizes were constructed to proceed the contrastive study of size effect of TiO_2_ HMS on photovoltaic performance of QDSSCs. To further explore if other QD systems are suitable for TiO_2_ HMS, the ZnS/PbS/CdS QDSSC (two SILAR cycles of PbS QDs) were also fabricated by a similar approach [29]. Each of the QDSSCs were repeatedly tested three times in order to guarantee their reproducibility.

## 3. Characterization

The surface morphological images of samples were captured by Quanta 450 FEG scanning electron microscopy (SEM, Hillsbro, OR, USA) equipped with an energy dispersive X-ray spectrometer (EDS) for surface elemental mapping analysis. The Tecnai G2 F20 transmission electron (TEM, Hillsboro, TX, USA) equipped with EDS components was employed to record the fine structure and elemental spot scanning of samples. The Nicolet_iS50 infrared spectrometer (IR, Thermo Fisher Scientific, CN) is used to analyze the functional groups of carbonaceous spheres. The crystal phase of TiO_2_ HMS was analyzed by D/MAX-2400 Rigaku X-ray diffraction (XRD, Rigaku, Japan). The Nitrogen adsorption-desorption isotherm and BET surface area analysis were conducted by Tirstar II 3020 to monitored the influence of size variation of TiO_2_ HMS on QDs loadings.

The J-V behaviors of QDSSCs assembled with ZnS/CdS@TiO_2_ HMS photoanodes were investigated by Oriel I-V test station (Newport, USA). The sunlight illumination with an intensity of 100 mW cm^−2^ was simulated by a solar simulator. The incident photo to the current conversion efficiency (IPCE) was monitored by a 150 W Xe lamp coupled with a computer controlled monochromator.

## 4. Results and Discussion

**Figure 1:**
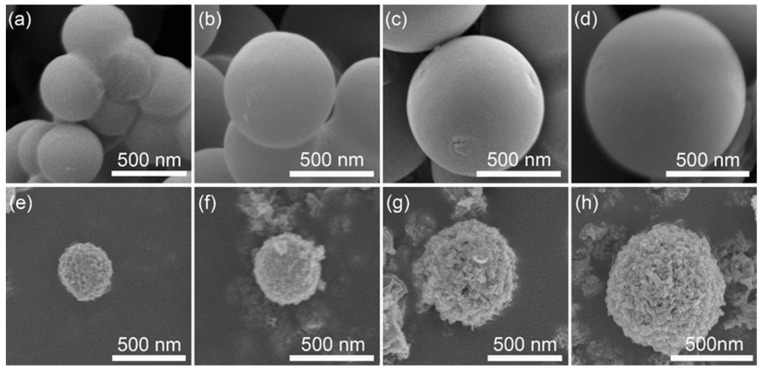
(**a**–**d**) SEM images of carbonaceous spheres (CS) with different sizes synthesized by tuning concentration of sucrose; (**e**–**h**) SEM images of TiO_2_ hollow microspheres (HMS) fabricated by different sizes of CS templates.

The size adjustability of CS is one of the most important factors in order to synthesize TiO_2_ HMS with different sizes. As a facile method, the size of CS can be easily varied by changing the concentration of sucrose aqueous solution when a hydrothermal process is conducted. Figure 1a–d displays the size changes of the resultant CS according to the increase of sucrose concentration. Apparently, the CS are well obtained by this hydrothermal process, and the size of CS increases from ~380 nm to ~1000 nm as the increment of sucrose concentration increases from 0.5 M to 1.5 M. Another key factor for the fabrication of TiO_2_ HMS is the adsorption ability of CS for Ti^4+^. According to a previous report [13,30], the hydrophilic groups that distribute on the surface of the CS template are favorable to the adsorption of metal ions. The IR spectrum of CS is presented in Figure S1 (Supplementary Figure S1), showing that the surface of CS contains OH, C–O, and C=O groups, which provide the sites for the anchoring of Ti^4+^).

Based on the CS template, TiO_2_ HMS are further obtained, which are shown in Figure 1e–h. In general, the surface of TiO_2_ HMS is rougher than their CS template, indicating that the TiO_2_ HMS are assemble by large numbers of nanoparticles. Moreover, the size of TiO_2_ HMS increases as larger CS template are employed, confirming that this method is feasible for the controllable synthesis of TiO_2_ HMS. A shrinkage phenomenon can be observed by a careful comparison between TiO_2_ HMS and their corresponding CS template. Taking Figure 1d,h as an example, the size of the CS template is ~1000 nm, however the product of TiO_2_ HMS is ~700 nm, of which ~30% shrinkage occurred after the formation of TiO_2_ HMS. Similar results are also observed in previous reports [18,19,31]. Therefore, TiO_2_ HMS with size distributions from ~330 nm, ~400 nm, ~560 nm, and ~700 nm are acquired by gradually increasing the CS template size.

**Figure 2:**
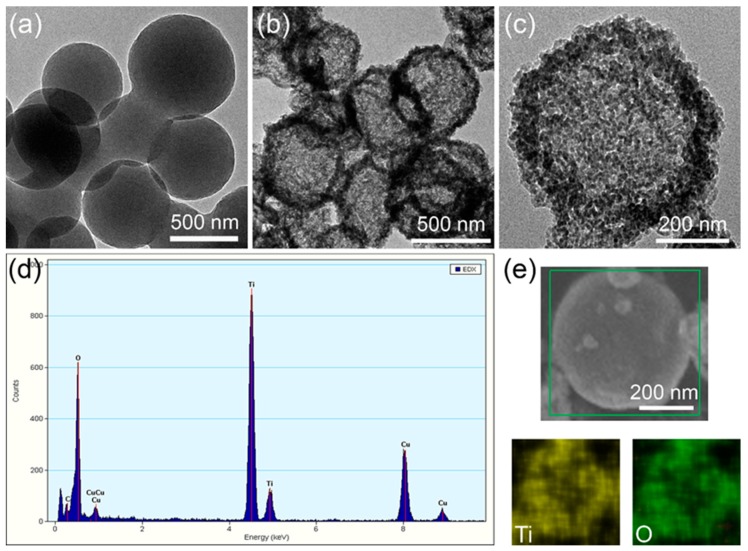
(**a**) TEM image of CS template; (**b**) TEM image of TiO_2_ HMS in low magnification; (**c**) magnified TEM image of TiO_2_ HMS; (**d**) EDS of TiO_2_ HMS; (**e**) SEM elemental mapping scan results of a single TiO_2_ HMS.

Although the spherical structure of TiO_2_ can be identified by SEM images from the resultant product, it seems that claiming the TiO_2_ structure belongs to the hollow microsphere is still insufficient with respect to direct proofs. Therefore, the TEM is used to present the fine structure of the products, which are shown in Figure 2a–c. Figure 2a shows the TEM image of the CS template, displaying a solid sphere structure. Obvious hollow spherical structures are obtained after soaking the CS template in Ti^4+^ solution and a subsequent annealing process, as shown Figure 2b, confirming that CS template method is a feasible approach to fabricate TiO_2_ HMS. Figure 2c presents a single TiO_2_ HMS in larger magnification, which shows that the shell of a hollow sphere is composed of a large number of nanoparticles, demonstrating that Ti^4+^ precursors adsorbed by the CS template were gradually turned into TiO_2_ nanoparticles and aggregated on the surface of the CS template during the annealing process, and finally led to the shell of a hollow microsphere. The EDS in Figure 2d evidenced the elemental composition of HMS, showing two sharp peaks which represent O and Ti elements, respectively. Further elemental mapping scan results from the SEM of HMS as shown in Figure 2e display that O and Ti elements are uniformly distributed on the surface of HMS, confirming the hollow microspherical structure is TiO_2_.

The crystal structure of TiO_2_ HMS is analyzed by XRD, which is shown in Figure S2 (Supplementary Figure S2). Several sharp diffraction peaks can be discerned in this XRD pattern, especially peaks that appeared around 2θ = 25.2°, 37.8°, 48.0°, 53.7°, and 71.4° which can be ascribed to the (101), (004), (200), (105), and (220) planes of the tetragonal anatase phase according to JCPDS Card No. 21-1272. The sharpening of diffraction reflections suggests their relative strong crystallinity due to the 500 °C calcination temperature used in the production of TiO_2_ HMS. As the SEM, TEM, EDS, and XRD analysis results showed, it can be concluded that anatase TiO_2_ HMS with different size distributions can be controllably fabricated by the CS template method.

**Figure 3:**
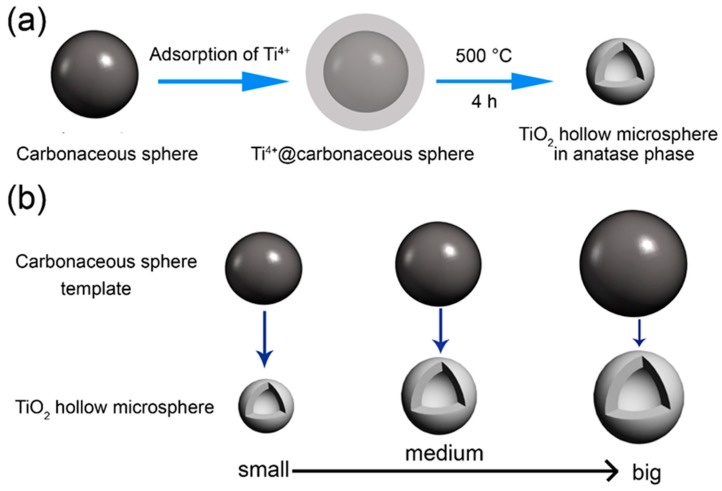
(**a**) The formation illustration of TiO_2_ HMS by CS template method; (**b**) the mechanism of size controllable synthesis of TiO_2_ HMS by CS template method.

The formation and size controllable synthesis mechanisms of anatase TiO_2_ HMS are illustrated in Figure 3 according to the experimental process and characterization results. The most important part in the fabrication process is the CS templates. As indicated by Figure S1, the surface of CS templates is rich with carboxyl and hydroxyl groups, which are beneficial to the adsorption of Ti^4+^ [13]. As presented in Figure 3a, initially the CS template is dispersed in Ti^4+^ precursors for 6 h to adsorb Ti^4+^ ions on its surface, leading to the formation of Ti^4+^@carbonaceous sphere. Subsequently, the carbonaceous sphere turns to CO_2_ during the annealing process. In the meantime, the Ti^4+^ ions adsorbed on the surface of the carbonaceous sphere are oxidized to anatase TiO_2_ nanoparticles which are assembled according to the shell of the hollow sphere, leading to the formation of TiO_2_ HMS. Furthermore, if different sizes of the CS template are selected, the size controllable synthesis of TiO_2_ HMS can be realized as illustrated in Figure 3b.

**Figure 4:**
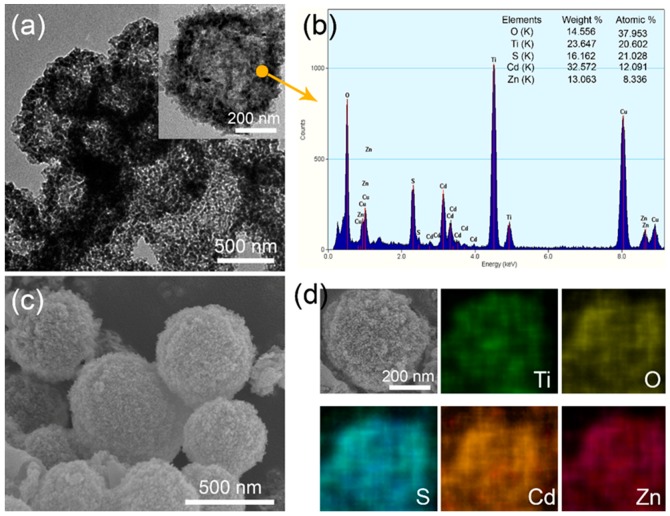
(**a**) TEM of ZnS/CdS@TiO_2_ HMS, the inset is magnified TEM of single ZnS/CdS@TiO_2_ HMS; (**b**) EDS of ZnS/CdS@TiO_2_ HMS from the selected spot; (**c**) SEM of ZnS/CdS@TiO_2_ HMS; (**d**) surface SEM elemental mapping of ZnS/CdS@TiO_2_ HMS.

Based on TiO_2_ HMS, the ZnS/CdS QDs have been anchored onto the surface by the SILAR method. The morphological variations of HMS have been revealed by TEM and SEM images. Figure 4a shows the TEM of ZnS/CdS@TiO_2_ HMS, displaying that the hollow spherical structure still preserves well but with a thicker thickness of the shell. With a magnified TEM in the inset of Figure 4a, it can be discerned that many small nanoparticles assembled on the shell of TiO_2_ HMS, making the shell’s thickness thicker than bare TiO_2_ HMS which is shown in Figure 2c; this implies that ZnS/CdS QDs are successfully anchored onto the shell of TiO_2_ HMS. The EDS result of a selected spot of ZnS/CdS@TiO_2_ HMS is shown in Figure 4b. Excepting the Ti and O elements contributed by the TiO_2_ HMS supporting architectures, other peaks including Zn, Cd, and S can be discerned in EDS, and it is worth noting is that the atomic ratio of (Zn+Cd): S is very close to 1:1, which indicates the successful decoration of ZnS/CdS QDs on TiO_2_ HMS. Similar phenomena can also be identified by the SEM image of ZnS/CdS@TiO_2_ HMS in Figure 4c. After sensitization with ZnS/CdS QDs, many smaller nanoparticles were covered on the surface of TiO_2_ HMS, which cannot be observed in SEM images of bare TiO_2_ HMS shown in Figure 1e–h. The SEM elemental mapping scan results on the surface of HMS in Figure 4d show that Zn, Cd, S, O and Ti elements are homogenously distributed on the surface of HMS, also confirming the formation of ZnS/CdS@TiO_2_ HMS. Hence, ZnS/CdS QDs sensitized TiO_2_ HMS with different sizes are investigated in contrast to find out the size effect of TiO_2_ HMS on the photovoltaic performance of ZnS/CdS QDSSCs.

**Figure 5:**
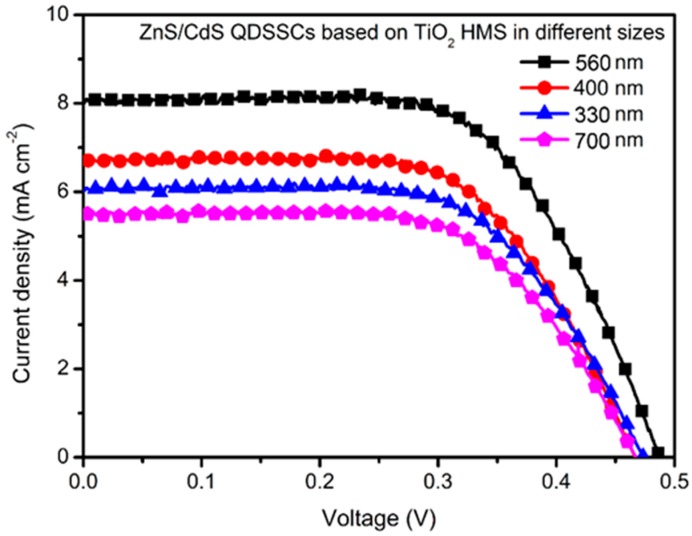
Current density-voltage (J-V) curves of QDSSCs based on ZnS/CdS@TiO_2_ HMS with size ~330 nm, ~400 nm, ~560 nm, and ~700 nm, respectively.

Figure 5 compares the best J-V curves of ZnS/CdS QDSSCs based on TiO_2_ HMS supporting architecture with different sizes (the best J-V curves were selected from three times repeated tests of these solar cells, See Supplementary Materials, Figure S4). The corresponding parameters of these solar cells, including open-circuit voltage (V_oc_), short-circuit current density (J_sc_), fill factor (FF), and power conversion efficiency (PCE), are summarized in Table 1. The ZnS/CdS@TiO_2_ HMS solar cell with a size ~330 nm shows a V_oc_ of 0.47 V, a J_sc_ of 6.23 mA cm^−2^, and a FF of 0.46, producing a PCE of 1.34%. The size increment of TiO_2_ HMS seems beneficial to improve photovoltaic performance. For example, the ZnS/CdS QDSSC based on TiO_2_ HMS with size ~400 nm presents a V_oc_ of 0.46 V, a J_sc_ of 6.86 mA cm^−2^, and a FF of 0.47, leading to PCE increases to 1.48%. The highest PCE of 1.83% among our series sample solar cells is achieved by TiO_2_ HMS with size ~560 nm, showing a V_oc_ of 0.49 V, a J_sc_ of 8.02 mA cm^−2^, and a FF of 0.47. However, a further increment of size to ~700 nm results in a decrease of PCE to 1.18%, which is mainly caused by the decrease of J_sc_ to 5.44 mA cm^−2^. In our series of QDSSCs, both the preparation method of ZnS/CdS QDs and the SILAR cycles on TiO_2_ HMS are the same; the only difference among these QDSSCs is the size of the TiO_2_ HMS supporting architecture, which may influence the QDs loadings and light utilization. Figure S3 (Supplementary Figure S3) shows the BET results of the smallest and largest TiO_2_ HMS of our series samples, it can be seen that the size increment of HMS from ~330 nm to ~700 nm did not lead to an obvious variation of BET surface area, meaning that the size of TiO_2_ HMS has little contribution to enhance QDs loadings. Therefore, it is believed that the size change of TiO_2_ HMS is a key factor responsible for the variation of photovoltaic performance, especially for J_sc_’s variation.

**Figure 6:**
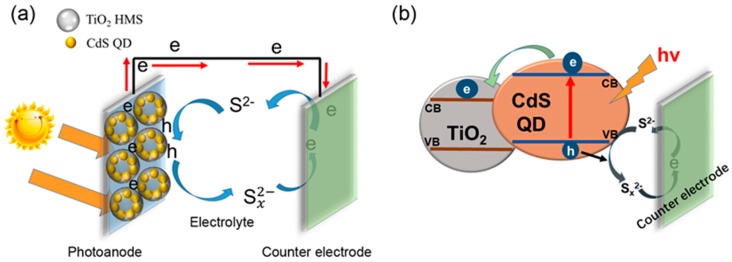
(**a**) The working model of ZnS/CdS@TiO_2_ HMS QDSSC; (**b**) the charges transport mechanism in ZnS/CdS@TiO_2_ HMS QDSSC.

Figure 6a,b presents the basic model and charge transport mechanism of ZnS/CdS@TiO_2_ HMS QDSSC, respectively. When the photoanode is illuminated, the CdS QDs are excited to generate electron-hole pairs. The electrons will be injected from CdS QDs into the conduction band of TiO_2_ HMS due to the band level alignment as shown in Figure 6b, and finally move to an out circuit, generating current. At the same time, the holes will be scavenged by a polysulfide electrolyte, and at the surface of the counterelectrode, electrons and holes combine together again, completing the cycle of charges. In comparison to the J-V parameters of our sample of QDSSCs, it is found that the J_sc_ increment is a key contributor to the enhancement of photovoltaic performance. According to the working model and charge transport mechanism proposed in Figure 6, we believe that the hollow spherical structure which may create multiple light reflection, and a size effect which may cause strong light scattering are the essence of the increment of J_sc_. According to the Mie theory and the Anderson localization of light [16,24,31], resonant scattering of light is anticipated to occur for the spherical particles with a size comparable to the incident light. In our situation, the hollow spherical structure can make the light reflected on shell of TiO_2_ HMS, and by controlling size of TiO_2_ HMS in appropriate range may result in stronger scattering effect, providing more opportunities for the photons to be absorbed by CdS QDs to enhance the light harvesting efficiency, and generate more electrons, eventually increasing the J_sc_.

**Figure 7:**
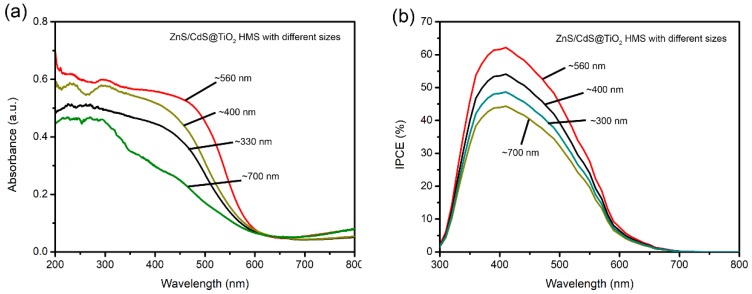
(**a**) UV-vis spectra of ZnS/CdS@TiO_2_ HMS with different sizes; (**b**) IPCE of ZnS/CdS@TiO_2_ HMS with different sizes.

In order to testify our claims, the UV-vis spectra of ZnS/CdS@TiO_2_ HMS with different sizes are recorded in Figure 7a. Two obvious phenomena are observed, one is that the onset of light absorption ~550 nm for all ZnS/CdS@TiO_2_ HMS photoanodes, indicating that the light absorption depend on ZnS/CdS QDs rather than TiO_2_ HMS. However, the absorbance of photoanodes seems to be influenced by sizes of TiO_2_ HMS, because the ZnS/CdS@TiO_2_ HMS with ~560 nm shows the higher absorbance than any other sizes of TiO_2_ HMS. As UV-Vis spectra indicated, the light absorption range of ZnS/CdS QDs is mainly below 550 nm, which means a strong light scattering may occur when the size of TiO_2_ supporting architecture is comparable with it. In our case, the ~560 nm TiO_2_ HMS is just in agreement with this wavelength to yield stronger light scattering and result in the enhancement of light harvesting efficiency, leading to the enhancement of absorbance. The incidental photon to current conversion efficiency (IPCE) of ZnS/CdS@TiO_2_ HMS with different sizes are monitored to further decipher the size effect of TiO_2_ HMS on photovoltaic performance, which are shown in Figure 7b. It can be found that the IPCE value depend greatly on the size of TiO_2_ HMS. Especially, the highest IPCE is achieved by ZnS/CdS@TiO_2_ HMS with size ~560 nm. Three factors including light harvesting efficiency (*LHE*), electron injection efficiency (*Φ*_ing_), and charge collecting efficiency (*η*_cc_) determine the IPCE value, as following equations [32]:

IPCE = *LHEΦ*_ing_*η*_cc_ (1)

*LHE* =1 − 10^−absorbance^ (2)

In our case, apart from light reflection at the shell of the hollow spherical structure, a stronger light scattering is caused by ~560 nm TiO_2_ HMS comparable to the wavelength of light absorbed by ZnS/CdS QDs, which enhances the light harvesting efficiency and contributes to the increase of IPCE. Furthermore, stronger light scattering lead to QDs absorbing more photons, and yield a higher absorbance to enhance the light harvesting efficiency, finally leading to high IPCE value and J_sc_. These results confirmed that the superior light scattering properties depend on the size effect of TiO_2_ HMS, and changing the size in an appropriate range to agree with wavelength of absorbed light is beneficial to enhance the light harvesting efficiency.

As previous reports have mentioned [33,34], the PbS QDs can help to absorb more photons and increase the J_sc_ further. The ZnS/PbS/CdS QD was applied to our ~560 nm TiO_2_ HMS to further improve photovoltaic performance and explore if the supporting architecture is suitable in other types of QDSSC. Figure S4 (Supplementary Figure S4) shows the best I-V test result of ZnS/PbS/CdS QDSSC based on ~560 nm TiO_2_ HMS, presenting a PCE of 2.73% with a significant increment of J_sc_ to 13.50 mA cm^−2^. This result demonstrated that a high efficient QDSSC is predictable by tailoring suitable QDs system in TiO_2_ HMS.

## 5. Conclusions

In summary, size controllable TiO_2_ HMS have been successfully synthesized by the CS template method. Based on TiO_2_ HMS with sizes of ~330 nm, ~400 nm, ~560 nm, and ~700 nm, the ZnS/CdS QDs sensitized TiO_2_ HMS solar cells have been constructed. The size effect of TiO_2_ HMS on photovoltaic performance of QDSSC has been investigated in contrast. It was found that with the same ZnS/CdS QDs, the photovoltaic performance, especially the J_sc_ of sample solar cells, depend greatly on the size of TiO_2_ HMS. In comparison, when the size of TiO_2_ HMS (~560 nm) is comparable to the light wavelength that ZnS/CdS QDs (~550 nm) can absorb, the best J_sc_ of 8.02 mA cm^−2^ and the highest PCE of 1.83% among the sample solar cells have been achieved. The UV-vis and IPCE analysis confirmed that a stronger light scattering generated by TiO_2_ HMS with ~560 nm is responsible for the enhancement of light harvesting efficiency, leading to the increment of J_sc_ and PCE. The further application of ~560 nm TiO_2_ HMS in ZnS/PbS/CdS QDSSC can improve the PCE to 2.73%, demonstrating the wide applicability of TiO_2_ HMS in QDSSCs. These findings may offer potential insights with respect to the design of TiO_2_ architectures for improving the photovoltaic performance of QDSSCs.

## Figures and Tables

**Table 1:** Size effect of TiO_2_ HMS on photovoltaic performance of ZnS/CdS QDSSC.

Size (nm)	V_oc_ (V)	J_sc_ (mA cm^−2^)	FF	PCE (%)
330	0.47	6.23	0.46	1.34
400	0.46	6.86	0.47	1.48
560	0.49	8.02	0.47	1.83
700	0.46	5.44	0.46	1.18

## Supplementary Materials

The following are available online at https://www.mdpi.com/1996-1944/12/10/1583/s1, Figure S1: The IR spectrum of carbonaceous spheres template, Figure S2: The XRD pattern of TiO_2_ hollow microspheres obtained by carbonaceous template method, Figure S3: The N_2_ adsorption-desorption isotherm curves of TiO_2_ HMS with different sizes, (a) ~330 nm, (b) ~700 nm, Figure S4: The J-V curves of QDSSC based on TiO_2_ HMS with different sizes, (a) ~330 nm TiO_2_ HMS, (b) ~400 nm TiO_2_ HMS, (c) ~560 nm TiO_2_ HMS, (d) ~700 nm TiO_2_ HMS; three times repeated tests were carried out on each QDSSC, and the best results are showed, Figure S5: The J-V curve of ZnS/PbS/CdS QDSSC based on ~560 nm TiO_2_ HMS.
