# Supplementary material for: The Size Effect of TiO2 Hollow Microspheres on Photovoltaic Performance of ZnS/CdS Quantum Dots Sensitized Solar Cell
Source: Materials (Basel). 2019 May 15;12(10):1583. doi: 10.3390/ma12101583 (PMC6566662; doi:10.3390/ma12101583)
Supplement: Supplementary file 1 [file materials-12-01583-s001.pdf]

materials

MDPI

Supplementary Materials

The Size Effect of TiO_2 Hollow Microspheres on Photovoltaic Performance of ZnS/CdS Quantum Dots Sensitized Solar Cell

Zhen Li [1,2], Libo Yu [1,*]

[1] Institute of Flexible Composite Materials, College of Chemistry and Chemical Engineering, Hexi University, Zhangye City 734000, Gansu Province, China; lizhen@hxu.edu.cn
[2] Key Laboratory of Hexi Corridor Resources Utilization of Gansu, Hexi University, Zhangye City 734000, Gansu Province, China
* Correspondence: yulibo@hxu.edu.cn; Tel.: +86-1569-364-8726

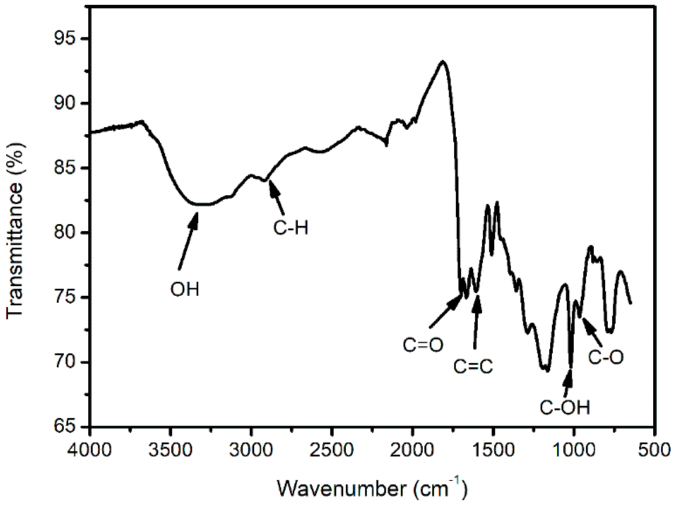

Figure S1. The IR spectrum of carbonaceous spheres template.

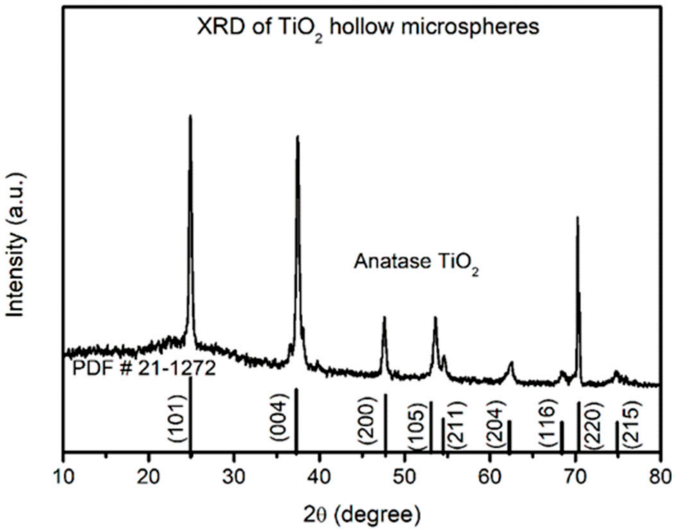

Figure S2. The XRD pattern of TiO_2 hollow microspheres obtained by carbonaceous template method.

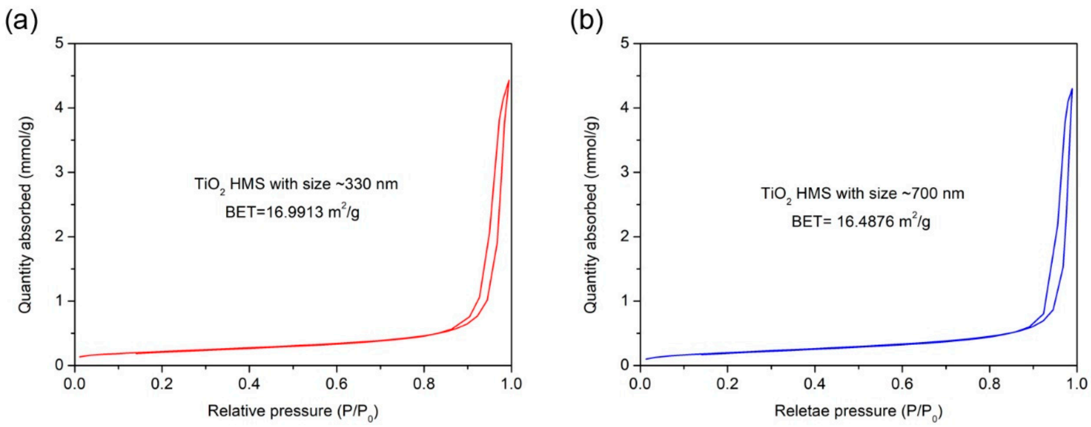

Figure S3. The N_2 adsorption-desorption isotherm curves of TiO_2 HMS with different sizes, (**a**) ~330 nm, (**b**) ~700 nm.

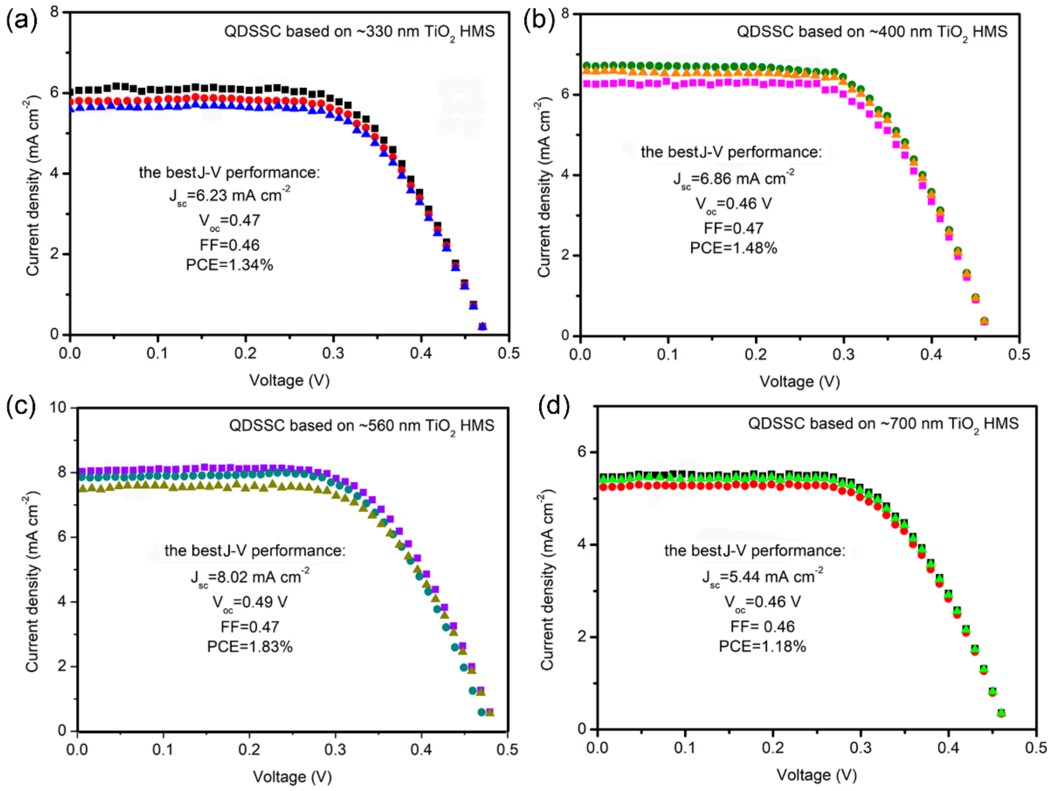

Figure S4. The J-V curves of QDSSC based on TiO_2 HMS with different sizes, (**a**) ~330 nm TiO_2 HMS, (**b**) ~400 nm TiO_2 HMS, (**c**) ~560 nm TiO_2 HMS, (**d**) ~700 nm TiO_2 HMS; three times repeated tests were carried out on each QDSSC, and the best results are showed.

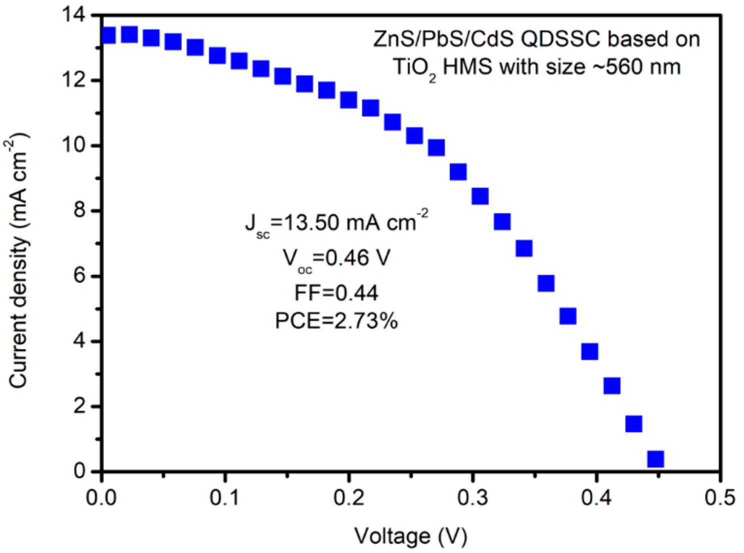

Figure S5. The J-V curve of ZnS/PbS/CdS QDSSC based on ~560 nm TiO_2 HMS.
